# Physically Triggered Morphology Changes in a Novel *Acremonium* Isolate Cultivated in Precisely Engineered Microfabricated Environments

**DOI:** 10.3389/fmicb.2017.01269

**Published:** 2017-07-14

**Authors:** Laura Catón, Andrey Yurkov, Marcel Giesbers, Jan Dijksterhuis, Colin J. Ingham

**Affiliations:** ^1^Hoekmine BV Utrecht, Netherlands; ^2^Leibniz-Institut DSMZ-Deutsche Sammlung von Mikroorganismen und Zellkulturen GmbH Braunschweig, Germany; ^3^Wageningen Electron Microscopy Centre, Wageningen University Plant Sciences Wageningen, Netherlands; ^4^Westerdijk Fungal Biodiversity Centre-KNAW Fungal Biodiversity Centre Utrecht, Netherlands

**Keywords:** growth on surfaces, mycelial organization, cultivation chips, fungi, simulated environments

## Abstract

Fungi are strongly affected by their physical environment. Microfabrication offers the possibility of creating new culture environments and ecosystems with defined characteristics. Here, we report the isolation of a novel member of the fungal genus *Acremonium* using a microengineered cultivation chip. This isolate was unusual in that it organizes into macroscopic structures when initially cultivated within microwells with a porous aluminum oxide (PAO) base. These “templated mycelial bundles” (TMB) were formed from masses of parallel hyphae with side branching suppressed. TMB were highly hydrated, facilitating the passive movement of solutes along the bundle. By using a range of culture chips, it was deduced that the critical factors in triggering the TMB were growth in microwells from 50 to 300 μm in diameter with a PAO base. Cultivation experiments, using spores and pigments as tracking agents, indicate that bulk growth of the TMB occurs at the base. TMB morphology is highly coherent and is maintained after growing out of the microwells. TMB can explore their environment by developing unbundled lateral hyphae; TMB only followed if nutrients were available. Because of the ease of fabricating numerous microstructures, we suggest this is a productive approach for exploring morphology and growth in multicellular microorganisms and microbial communities.

## Introduction

Microfabrication (microelectromechanical systems – MEMS, lab on a chip) techniques offer very precise methods of building habitats for microorganisms. Parameters such as texture, surface chemistry, porosity, and the dimensions of separate or interconnected spaces can be systematically varied, often within a single device. This set of techniques has been applied to the growth of bacteria and fungi in constrained conditions and allows development, dispersal and decision-making to be tested in controlled environments. MEMS fabrications and other microengineered devices have been used to look at the growth of fungi in new ways (Crane et al., 2014). Microfluidics has been used to look at spore outgrowth in the pathogenic fungus *Cryptococcus neoformans* and to separate the role of carbon and nitrogen sources in germination (Barkal et al., 2016). Extremely precise methods have been devised to measure growth in *Aspergillus niger* using miniaturized cantilever-based sensors (Maloney et al., 2014). In another approach to manipulating fungi, spores of *Aspergillus fumigatus* have been contact-printed in a highly multiplexed and precise way (Ingham et al., 2010).

One approach to cultivation at the microcolony level has been to create arrays of microwells (7 μm diameter or larger) with a porous ceramic (porous aluminum oxide, PAO) base (Ingham et al., 2007). PAO is a self-ordering material that results from the anodization of aluminum under acidic conditions to form a highly porous filter with low background to many imaging techniques and an extremely flat and inert surface (Hoar and Mott, 1959; Ingham et al., 2012b; Lee and Park, 2014). PAO has been shown to be an effective substrate for the growth and imaging of *Candida* and *Aspergillus* spp. and the action of antifungal agents on them (Ingham and Schneeberger, 2012; Ingham et al., 2012a). Culture on PAO-derived disposables is a flexible method, allowing new formats to be rapidly fabricated by redesigning the shadow mask used to specify the dimensions and spacing of the compartments (microwells). Such cultivation chips have been used in environmental screenings and coupled with flow cells to look at fluctuating gene expression patterns in microorganisms confined in microwells (Hesselman et al., 2012).

Fungi can sense physical parameters of their environment and respond with changes in their directed growth or development. This sensitivity includes pathways of mechanosensing that result in changes in growth including differentiation and host invasion (Bahn et al., 2007; Kumamoto, 2008). One example of this is the *Uromyces* (rust) germ tube, which exhibits thigmotropism: the fungus regulates the directional growth of germ tubes and induces their development into appressoria initiated by topographical signals on the host surface (Wynn, 1976; Hoch et al., 1987). Yeasts are also highly sensitive to their physical environment, this being an important factor in dimorphism between filamentous and unicellular forms (Brand et al., 2014).

The environment of many microorganisms is complex, spatially heterogeneous and subject to fluctuations in nutrients, other organisms and physical parameters such as light, temperature and humidity (Weibel et al., 2007; Kumar et al., 2013). Typically, microbiologists study fungi in the natural environment and also in situations very distant from the natural situation, such as monoculture on an agar plate. Microfabricated environments can be constructed with very precisely defined physical and chemical parameters (Ma et al., 2014) to mimic or vary specific environments which can be closely monitored and varied in a controlled way. Topology, access to nutrients and the degree of interactivity between microorganisms can be systematically varied. Such cultivation chips have been used to isolate new species, including previously uncultivated microorganisms (Kaeberlein et al., 2002; Ingham et al., 2007; Lok, 2015). Multiple environments can be placed on a single disposable. Linked microenvironments can be used to study microbial competition and dispersal, simulating many environments including the soil (Keymer et al., 2006; Wessel et al., 2013; Stanley et al., 2016).

We report the isolation of a new species of filamentous fungus of the genus *Acremonium* taken from a marine sponge, using miniaturized disposable Microbial Culture Chips (MCC). The *Acremonium* isolate showed great sensitivity to the topology of its environment, being triggered to create parallel bundles of hyphae in a precisely engineered physical environment. The factors influencing this unusual developmental process were investigated using a series of micron-scale growth environments.

## Materials and Methods

### Design, Fabrication, and Preparation of Culture Chips

**FIGURE 1:**
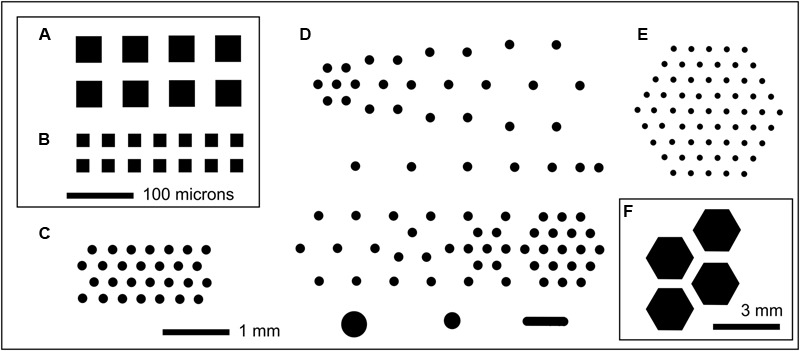
Selected examples of MCC layouts showing well patterning in black. In each case a small fraction of an 8 mm × 36 mm culture chip is shown. **(A)** MCC40.10 with 40 μm × 40 μm compartments (black) with 10 μm deep wells with PAO at the base; each well is separated by 30 μm wide walls with orthogonal spacing repeated over the entire 8 mm × 36 mm chip. **(B)** MCC20.10, orthogonal arrangement of 20 μm × 20 μm square microwells with 20 μm wide walls. Scale bar (100 μm) applies to both **(A,B)**. **(C)** MCC180.10 (also MCC180.40) with hexagonal array of 180 μm diameter compartments separated by 160 μm width walls, with the wells 10 or 40 μm deep, respectively. **(D)** MCC180.10VAR with variably spaced 180 μm diameter wells (10 μm deep) plus a small number of larger compartments (bottom row). This block of 6 mm × 6 mm is repeated over the surface of the 8 mm × 36 mm MCC. **(E)** MCC100.10HEX with 100 μm diameter compartments with 200 μm spacing. Each hexagonal array is separated from adjacent hexagonal arrays by 0.4 mm, with this arrangement repeated over the surface of the MCC [shown in **(F)**, where the blocks of wells can be seen]. MCC50.10HEX are similar to MCC100.10HEX but with 50 μm diameter compartments separated by 250 μm wide walls.

Culture chips (MCC) were used to screen microorganisms grown to microcolonies (Ingham et al., 2007). MCC contained arrays of microwells with a PAO base (8 mm × 36 mm, 60 μm thick, 40% porosity, 20 to 200 μm pore diameter) acting as a sterile filter. MCC were fabricated by patterning the wall material (Ordyl 300 film, Elga, Italy) using photolithography and then applying it to 8 mm × 36 mm strips of PAO arrayed on a silicon wafer. Walls were patterned by photolithography of 10 or 40 μm thick Ordyl 300 film with development according to the manufacturer’s protocols. The resulting perforated and processed material was heat/pressure applied to the PAO. Optionally, gold or platinum (5 to 20 nm) was used to sputter coat the upper surface of the MCC. The MCC designs manufactured are summarized in **Figure 1** and **Table 1**. Compartment geometry was usually round or square with a hexagonal or orthogonal arrangement, respectively. All MCC except the MCC180.10VAR and MCC100HEX.100 had the same arrangement of wells (well size, wall width, compartment width, and spacing) over the entire surface. The MCC180.10VAR had repeating blocks (6 mm × 6 mm) containing 180 μm diameter wells but with variable spacing between the wells plus a small number of larger wells (**Figure 1**). Additionally, MCC180.10 and MCC180.40 had regions at the edge of the chip where the Ordyl wall bordered planar, porous PAO – i.e., functioned as a step of 10 or 40 μm in height with the base being PAO. The patterned wall material (Ordyl film with holes) used to manufacture the MCC was also applied directly to the agar surface to create microwells without the PAO base.

**Table 1:** Summary of cultivation chips fabricated.

Chip type	Compartment size (μm)^a^	Wall height (μm)	Wall width (μm)^b^	Compartment arrangement	No. compartments per chip	Reference
MCC7.10	7 (square)	10	10	Orthogonal	1,000,000	Ingham et al., 2007
MCC20.10	20 (square)	10	10	Orthogonal	320,000	Ingham et al., 2007
MCC20.10	20 (square)	10	20	Orthogonal	180,000	Ingham et al., 2007
MCC40.10	40 (square)	10	20	Orthogonal	80,000	Ingham et al., 2007
MCC30.10	30 (round)	10	30	Orthogonal	80,000	This work
MCC50.10HEX	50 (round)	10	250	Hexagonal	6,000	This work
MCC100.10HEX	100 (round)	10	200	Hexagonal	6,000	This work
MCC100.20	100 (square)	10	20	Orthogonal	20,000	Ingham et al., 2007
MCC150.20	150 (square)	10	20	Orthogonal	10,000	Ingham et al., 2007
MCC180.10	180 (round)	10	160	Hexagonal	4,500	Hesselman et al., 2012
MCC180.40	180 (round)	40	160	Hexagonal	4,500	Hesselman et al., 2012
MCC180.10VAR	180 (round)^c^	10	Variable	Variable	500	This work
MCC300.10	300 (round)	10	300	Hexagonal	900	This work
MCC500.10	300 (round)	10	500	Hexagonal	400	This work
MCC000.10	None	N/a	N/a	N/a	Single edge	This work
MCC000.40	None	N/a	N/a	N/a	Single edge	This work

*^a^Width for square compartments, diameter for round. ^b^Minimum distance between adjacent compartments. ^c^Chip design contains other compartment sizes in addition to 180 μm (see **Figure 1**).*

### Modification of Culture Chips and PAO

Platinum was sputtered over MCC or PAO to create a 10 nm thick layer over both PAO and wall material (Ingham et al., 2007). Sigmacote (Sigma, Netherlands) was applied to MCC or PAO to block the penetration of culture media through selected regions of the porous ceramic by rendering the pores hydrophobic (Ingham et al., 2010). PAO pores were physically blocked by applying graphite conductive adhesive (154 from EMS NL) to the underside of MCC and allowing it to dry before washing the chip in distilled water and sterilizing. Poly-L-lysine was also applied to PAO and MCC as previously described (Ingham et al., 2010).

### Alternative Versions of PAO

A variety of versions of porous alumina were obtained to test effects of surface texture and treatment on fungal growth. Standard PAO (asymmetric with 200 and 20 nm pore size, 40% porosity) was obtained from General Electric (Germany) under the trade name Anopore. Other types of PAO were obtained from Smart Membranes (Germany). The PAO was used without surface treatment, or with from 5 to 25 nm platinum sputtered over the surface, or with regions made hydrophobic with hexamethyldisilazane treatment as previously described (Ingham et al., 2010).

### Isolation and Cultivation of *Acremonium* sp.

**FIGURE 2:**
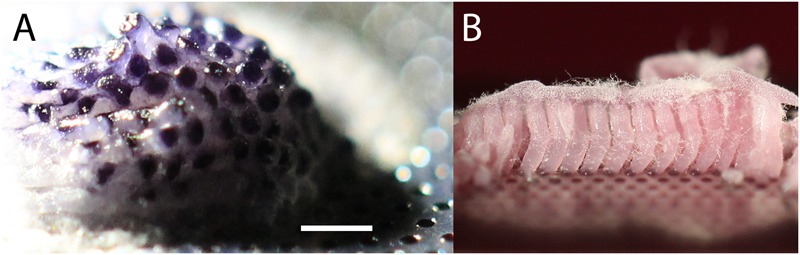
**(A)** Original isolate on MCC after growth for 3 weeks at room temperature with culture chip placed on ASWCLN agarose containing nigrosine. Nigrosine from the agarose has been taken up by the colony, and the hexagonal patterning of the 180 μm diameter wells is reflected in selective staining of the colony. The colony extends >3 mm above the surface of the MCC. **(B)** Subculture from A onto ASWCLN containing a pink food dye on a second MCC, inoculating multiple adjacent wells. The dye is again taken up into the colonies, which grow as separate hyphal bundles about 3 mm above the surface of the MCC. The white fluffy material at the top of the pink hyphal bundles is dispersed hyphae and phialides. The scale bar in **(A)** indicates 0.7 mm when applied to **(A)** and 1 mm for **(B)**.

A marine sponge (*Suberites ficus* – Sea Orange) was collected from the coast of Roscoff (France) by diver and stored in an aquarium and subsequently at -80°C. Sponge samples were homogenized and soaked in equal volumes of broth (1 g/l yeast extract, 10 g/l artificial sea salts). Culture was on sterile MCC (**Figures 1, 2** and **Table 1**), on PAO strips placed on agar, or direct on agar. In all cases the underlying medium was ASWCL agar, which contained 5 g/l κ-carrageenan, 5 g/l yeast extract, 15 g/l agar, and 1% artificial sea salts (Sigma, Netherlands), unless specified otherwise. Where appropriate, pigments (nigrosine 20 mg/l, Dextran Blue 100 mg/l, food dyes as below) were added before autoclaving. When nigrosine was added this medium was called ASWCLN. Fluorescent dyes (fluorescein, rhodamine) or antifungal (voriconazole) or other bioactive compounds [sodium arsenate, carbonylcyanide 3-chrorohydrazone (CCCP), cycloheximide, sodium azide] were added to growth media after filter sterilization at concentrations indicated in individual experiments. All dyes and bioactive agents were obtained from Sigma except for orange food gel (Americolor Orange, Americolor, United States) and red ink (Ecoline, Royal Talens, Netherlands), both used at 400 μl/l of growth medium. Incubations were at 20°C from 1 day to 1 week under aerobic conditions. Spores were isolated by filtration of cultures from agar plates through non-absorbent cotton wool. Fungal strains used are summarized in **Table 2**.

**Table 2:** *Acremonium* strains screened for production of TMB.

*Acremonium* strain	Culture conditions	Source	Strain details
*Acremonium* sp.	ASWCLA	This work	Isolated from marine sponge, Roscoff, France, in 2013
*Acremonium hennebertii*	ASWCLA	Centraalbureau Schimmelcultures (CBS), Utrecht, Netherlands	CBS 768.69
*Acremonium handsfordii*	ASWCLA	CBS, Utrecht, Netherlands	CBS 384.96
*Acremonium implicatum*	ASWCLA	Oded Yarden	OY12307
*Acremonium* sp.	ASWCLA	Oded Yarden	OY36707
*Acremonium* sp.	ASWCLA	Oded Yarden	OY1707
*Acremonium* sp.	ASWCLA	Oded Yarden	OY34007

Microbial Culture Chips for fungal culture were sterilized by high intensity UV for 20 min (Novascan, PSD Digital Ozone System). PAO was sterilized by baking at 200°C for 1 h or by high intensity UV. Chips or PAO were placed on agar nutrient media and inoculated with spores of the fungus in 3 μl aliquots by pipetting or with a sterile sable paint brush. MCC were moved and strained as previously described (Ingham et al., 2007). Microcolonies were transferred between chips and agar using a fine toothpick targeted by low power microscopy.

### Molecular Identification and Phylogenetics of Isolated Strain

DNA was extracted from pure culture of the fungus on sterile MCC by the method of Peláez et al. (1996). Amplifications of the internal transcribed spacer (ITS) region were performed with primer pair ITS1/ITS4 (White et al., 1990). D1/D2 domains of the large-subunit (LSU) rRNA gene were amplified with the primer pair LR0R/LR5 (Vilgalys and Hester, 1990). Two fragments of the β-tubulin gene were amplified with primer pairs Bt1a/Bt1b and Bt2a/Bt2b (Glass and Donaldson, 1995).

DNA was amplified in 50 μl PCR mixtures containing the following final concentrations or total amounts: 100 ng DNA, 1× Q5 reaction buffer, 200 μM dNTPs, 0.5 μM forward primer and 0.5 μM reverse primer, and 0.02 U/μl Q5 High-Fidelity DNA Polymerase (New England BioLabs). All reagents were combined and heated at 98°C for 30 s. Thirty-five cycles of PCR were then performed by using 98°C for 10 s, 50°C for 30 s, and 72°C for 30 s, followed by 72°C for 2 min. The amplified fragments were purified and sequenced at GATC Biotech (Cologne, Germany). The partial sequences were assembled manually and a consensus sequence was generated by using the SeqMan program 7.0.0 (DNASTAR, Madison, WI, United States).

Sequences of the LSU rRNA gene (D1/D2 domains) were aligned with MAFFT server (current version 7) using the FFT-NS-i option (Katoh et al., 2005). The phylogenetic analysis of LSU rRNA gene sequences was done with MEGA 6 (Tamura et al., 2013) using the Maximum-Likelihood algorithm and the Kimura 2-parameter evolutionary model. Evolutionary rates among sites were modeled using the Gamma distribution. Gaps were treated as missing data. Concatenated analysis of LSU rRNA gene (D1/D2 domains), ITS region and β-tubulin genes was performed using the dataset TreeBASE S14232 published by Giraldo et al. (2014). Sequences of the *Acremonium* isolate were aligned manually within the aforementioned alignment. Phylogenetic relationships were inferred by the maximum likelihood (ML) method based on the general time reversible (GTR) model with RaxML (version 8.0.26) using raxmlGUI 1.31 (Silvestro and Michalak, 2012) and the GTRGAMMAI option with 1000 rounds of bootstrap replicates.

### MIC_50_ Determinations

MIC_50_ (minimal inhibitory concentration of an antimicrobial compound required to inhibit growth by 50%) determinations were made on ASWCL agar, scoring the concentration that inhibited growth by at least 50%. CCCP, voriconazole, cycloheximide, and arsenate used in MIC determinations were all obtained from Sigma.

### Tracking Hyphal Growth

In order to visualize what parts of the templated structures/hyphal bundles were growing, colored particles were applied to facilitate imaging hyphal extension. The *Acremonium* isolate was cultured on MCC180.10VAR for 2 to 5 days and then lightly dusted with colored particles (3 to 10 μm colored beads (Polysciences, Germany) or non-water-soluble artist’s pigments (chalk green, ultramarine blue; Swaak, Netherlands). These particles adhered stably to the hyphal masses. Imaging was done periodically, over a period of 3 to 24 h, with a Canon EOS-650 digital SLR camera mounted on an Olympus BX-41 microscope (see below). Images were compiled in ImageJ v1.45S (Schneider et al., 2012) using the particle tracker plug-in to quantify changes in distribution of the pigmented areas and therefore identify growing regions.

### Imaging

Culture chips and agar plates were imaged using an Olympus BX-41 equipped with ×4 and ×10 lenses (total magnification ×40 and ×100, respectively) with side illumination using a white LED (50 W, Edmund Optics, United Kingdom). Alternatively, a NIKON SMZ25 binocular microscope equipped with a Plan Apo objective was used. In the latter case, up to 30 pictures were taken at different focus heights in the NIKON software and stacked into Adobe Photoshop with autoblend. Imaging of fluorescence (rhodamine, fluorescein, Fun-1) was as previously described (Ingham et al., 2007). Images were captured using a Canon EOS-650 digital SLR camera mounted on the microscope or equipped with a 100 mm EF macro lens. Hyphae and spores were mounted on microscope slides and imaged at high magnification using ×20 and ×50 lenses (total magnification ×200 and ×500). ImageJ version 1.45S (Schneider et al., 2012) was used to assist in measuring distances between hyphal branching and spore numbers from TIFF images.

### Dye Transport Studies

To visualize the transport of dyes along hyphal bundles, the fungus was first grown on MCC180.10 or MCC180.10VAR placed on ASWCL agar lacking any pigments. The MCC were then moved to new plates containing dyes and/or metabolic inhibitors as previously described (Ingham et al., 2007). The progress of fluorescent or fluorogenic dyes (rhodamine 0.01%, fluorescein 0.01%) from well to tip was monitored by fluorescence microscopy. Quantification was performed using calibrated fluorescent beads (Polysciences, Germany) using ImageJ.

### Scanning Electron Microscopy (SEM)

Samples of fungi on MCC, PAO or agar were glutaraldehyde fixed and treated with osmium tetroxide *in situ* as previously described (Ingham et al., 2012a). During ethanol dehydration, the *Acremonium* sp. detached from the PAO or MCC, allowing it to be placed on SEM stubs at different orientations to image different aspects of the colony. Imaging was as previously described (Ingham et al., 2012a,b).

### Cryo-SEM

The *Acremonium* isolate was cultured in microwells of MCC as described above. Different stages of growth and superstructure development were selected using a stereomicroscope and gently broken with a surgical blade. Microwells were transferred together with a piece of agar to a copper cup for snap-freezing in nitrogen slush. Agar blocks were glued to the copper surface with frozen tissue medium (KP-Cryoblock, Klinipath, Duiven, Netherlands). Samples were examined in a JEOL 5600LV scanning electron microscope (JEOL, Tokyo, Japan) equipped with an Oxford CT1500 Cryostation for cryo-SEM. Electron micrographs were acquired after sputter-coating using a gold target for three times during 30 s. Micrographs were taken at 5 kV and processed with Adobe Photoshop CS5.

### Culture on Sponge Surfaces

Samples of the sponge from which the *Acremonium* sp. was isolated were cut with a razor blade into 1 cm × 1 cm sections and sterilized by soaking in 50% ethanol for 1 h followed by 1% (w/v) Sea Salts (Sigma, Netherlands) three times for 1 h to remove the ethanol. The sponge samples were placed in a Petri dish and agar containing 1% (w/v) Sea Salts poured around the sponge, leaving both cut and uncut surfaces exposed. The surface was inoculated with the *Acremonium* isolate and the Petri dishes cultivated for 1 week at 22°C. The identity of fungi growing on the sponge surface was verified by LSU sequencing. Sectioning the sponge sample followed by staining with the dye Fun-1 (Life Technologies, Netherlands) and imaging by fluorescence microscopy was used to assess the penetration of the sponge by the fungus.

## Results

### Isolation of a Novel Fungus on MCC

A high-throughput screen (12,000 microcolonies) was made of microorganisms extracted from sponge samples from shallow waters off the coast of Roscoff, France, using MCC180.10 culture chips placed on plates of ASWCLN agar, containing the black dye nigrosine. MCC were used because they limited the spread of motile or rapidly growing microbes whilst allowing high quality imaging. The MCC used had a hexagonal arrangement of circular culture wells (180 μm diameter, 10 μm deep with a PAO base) (**Figure 1** and **Table 1**). An unusual colony was observed (**Figure 2A**) after 2 weeks incubation at 20°C. The colony had overgrown the wells and was 3 mm diameter and 2 mm high. The notable feature was that the hexagonal microwells appeared to be visible on the upper surface of the colony, suggesting selective uptake of the nigrosine from the agar through the pores of the aluminum oxide. When this colony was transferred onto ASWCLN agar, a fungus grew with a flat network of horizontal hyphae and abundant phialides; this colony morphology bore little resemblance to that of the colony on the original MCC. Repeated restreaking on agar (8 passages) resulted in the same morphology. However, inoculation from agar back onto MCC180.10 at any stage gave a colony structure similar to the original morphology of the isolate (**Figure 2B**). We refer to these structures as “templated mycelial bundles” (TMB). The TMB grew out of the MCC wells with the same diameter as the wells (±15%) and absorbed dyes from the culture medium. The dye-absorbing structures were atypical: most microorganisms (archaea, terrestrial fungi, prokaryotes) cultured on the MCC until overgrowth would form laterally spreading colonies that no longer conformed to or were templated by the pattern of underlying wells. When the isolate was cultured on a simple strip of PAO, without microwells, the colony morphology resembled culture directly on agar and lacked TMB.

### Identification and Phylogenetics of the Fungal Isolate

**FIGURE 3:**
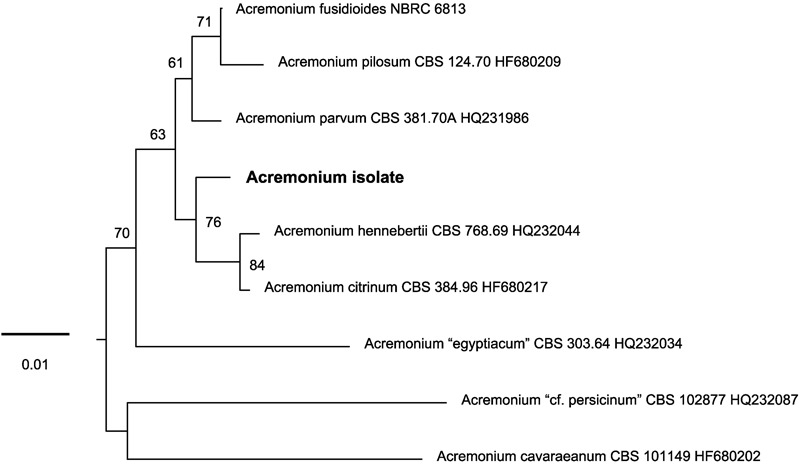
Unrooted phylogenetic tree inferred with maximum-likelihood analysis of the LSU rRNA gene (D1/D2 domains) showing the placement of the *Acremonium* isolate in the *Acremonium fusidioides* clade. The numbers given on branches are frequencies (>50%) with which a given branch appeared in 1000 bootstrap replications. The scale indicates the number of expected substitutions accumulated per site.

**FIGURE 4:**
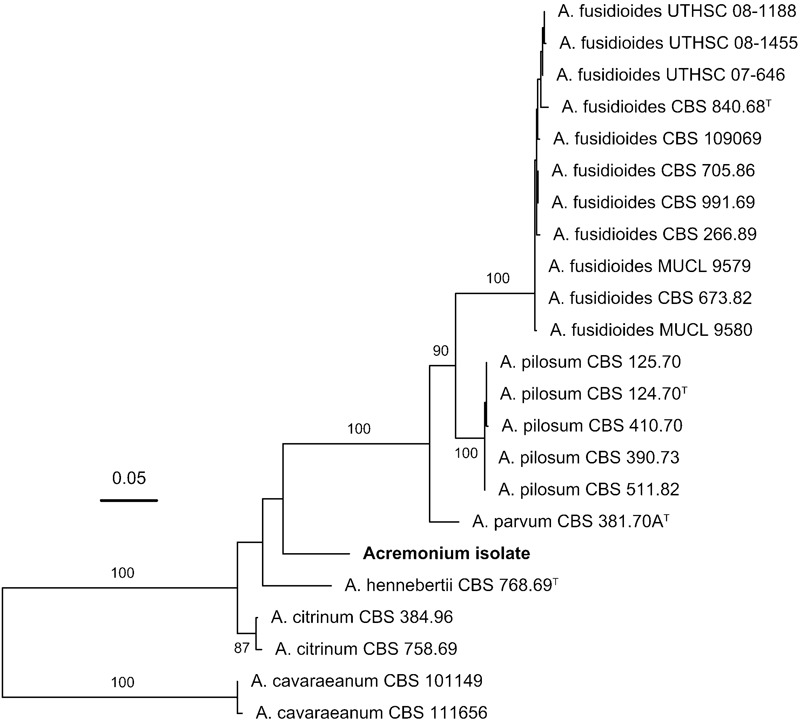
Phylogenetic placement of the *Acremonium* isolate in the *Acremonium fusidioides* clade *sensu* Summerbell et al. (2011) inferred with maximum-likelihood analysis of the concatenated alignment of the LSU rRNA gene (D1/D2 domains), ITS region and β-tubulin sequences. The numbers given on branches are frequencies (>75%) with which a given branch appeared in 1000 bootstrap replications. The scale indicates the number of expected substitutions accumulated per site.

To test the proposition that the same microorganism was being cultured on agar, PAO and MCC, DNA was extracted and ribosomal (SSU, ITS, LSU) and protein-coding (β-tubulin) gene fragments were sequenced. Nucleotide sequences of the isolate from all three environments (PAO, agar, culture chips) were identical. Comparison with sequences in public databases (NCBI GenBank and MycoBank) showed the closest matching with species comprising the *Acremonium fusidioides* clade (Summerbell et al., 2011). The taxonomy and phylogeny of this group has been recently reviewed by Summerbell et al. (2011) and Giraldo et al. (2014). Topology of the phylogenetic trees obtained in the present study was in agreement with the previous studies. Results of the phylogenetic analyses showed that the *Acremonium* isolate is only distantly related to any hitherto described species (Summerbell et al., 2011; Giraldo et al., 2014) (**Figures 3, 4**). Additionally, the isolate shared morphological characters distinguishing species in this complex such as elongated fusiform conidia arranged in chains. Screening of other *Acremonium* species (**Table 2**) on MCC180.10 did not reveal TMB. However, *Acremonium hennebertii* CBS 768.69 showed imperfect TMB after 6 days.

### Electron Microscopy of Developing Hyphal Bundles on MCC180.10

**FIGURE 5:**
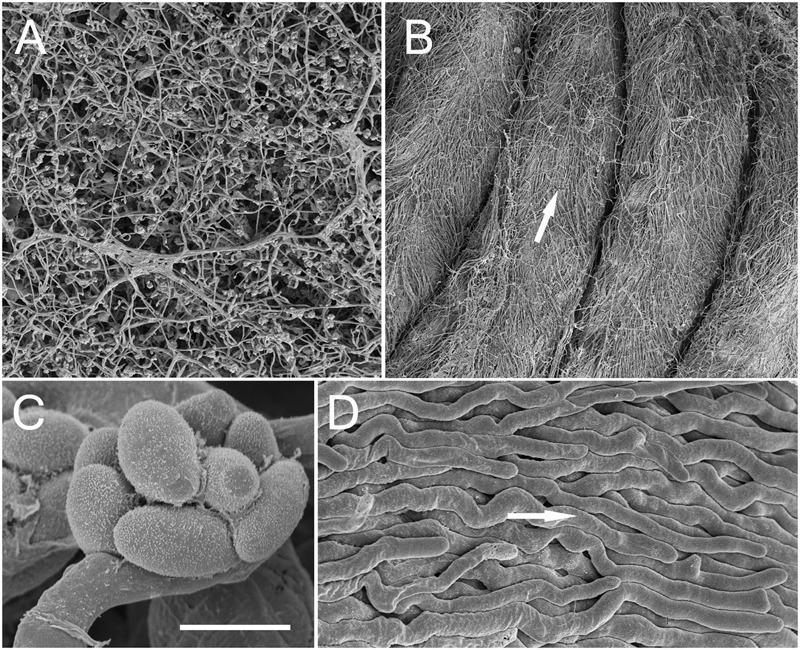
Comparison by SEM of the *Acremonium* sp. under different growth conditions. **(A)** Hyphal networks on unstructured PAO (the morphology growth directly on ASWCLN agar was similar). **(B)** TMB cultured in MCC180.10 then removed during fixation. White arrow shows direction of growth from base to tip. **(C)** Spores organized into a philiade; taken from PAO but similar when grown on agar or MCC180.10. **(D)** Detail of parallel bundles of hyphae from a TMB, as shown in **(B)**; arrow as **(B)**. Scale bar **(C)** indicates 20 μm when applied to **(A)**, 170 μm for **(B)**, 2 μm for **(C)** and 5 μm for **(D)**.

**FIGURE 6:**
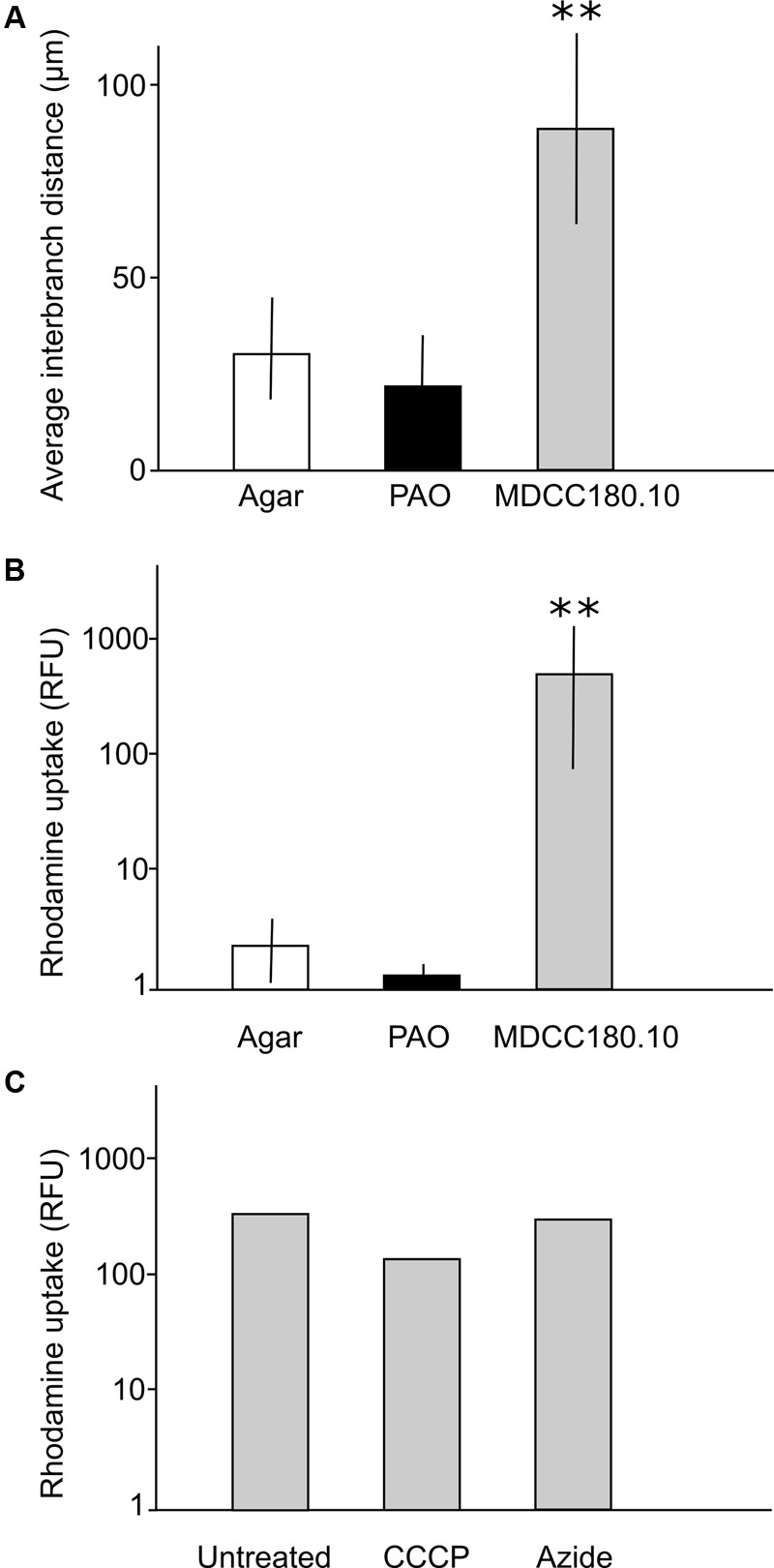
Quantification of differences between growth and dye uptake of *Acremonium* sp. on agar, PAO and MCC180.10. **(A)** Interbranch distance measured from scanning electron micrographs; *n* = 30; mean ± SD. ^∗∗^Indicates a significant difference in mean interbranch difference for TMB compared to the interbranch distance measured from mycelia grown on agar (Student’s *t*-test, *P* < 0.05). No significant difference was found in branching between the fungus grown on agar and unstructured PAO in either set of experiments (*P* > 0.5). **(B)** Quantification of rhodamine uptake (relative fluorescence units) for cultures grown on ASWCL containing 0.01% (w/v) rhodamine. *n* = 5; mean ± SD. ^∗∗^Indicates a significant difference in rhodamine uptake by TMB compared to the dye uptake by mycelia grown on agar (Student’s *t*-test, *P* < 0.05). No significant difference was found in dye uptake between growth on agar and unstructured PAO in either set of experiments (*P* > 0.5). **(C)** Quantification of rhodamine uptake for TMB formed in MCC180.10 on agar without rhodamine, then moved to new plates containing 0.01% (w/v) of the dye, with or without metabolic inhibitors, then incubated for 6 h before quantification. Inhibitors were present in the agar at a concentration sufficient to inhibit further growth. CCCP, 50 μM carbonyl cyanide *m*-chlorophenyl hydrazine; Azide; 50 μM sodium azide. There was no significant difference in dye uptake in these experiments comparing the untreated sample with either the azide or CCCP treatments (Student’s *t*-test, *P* > 0.1).

**FIGURE 7:**
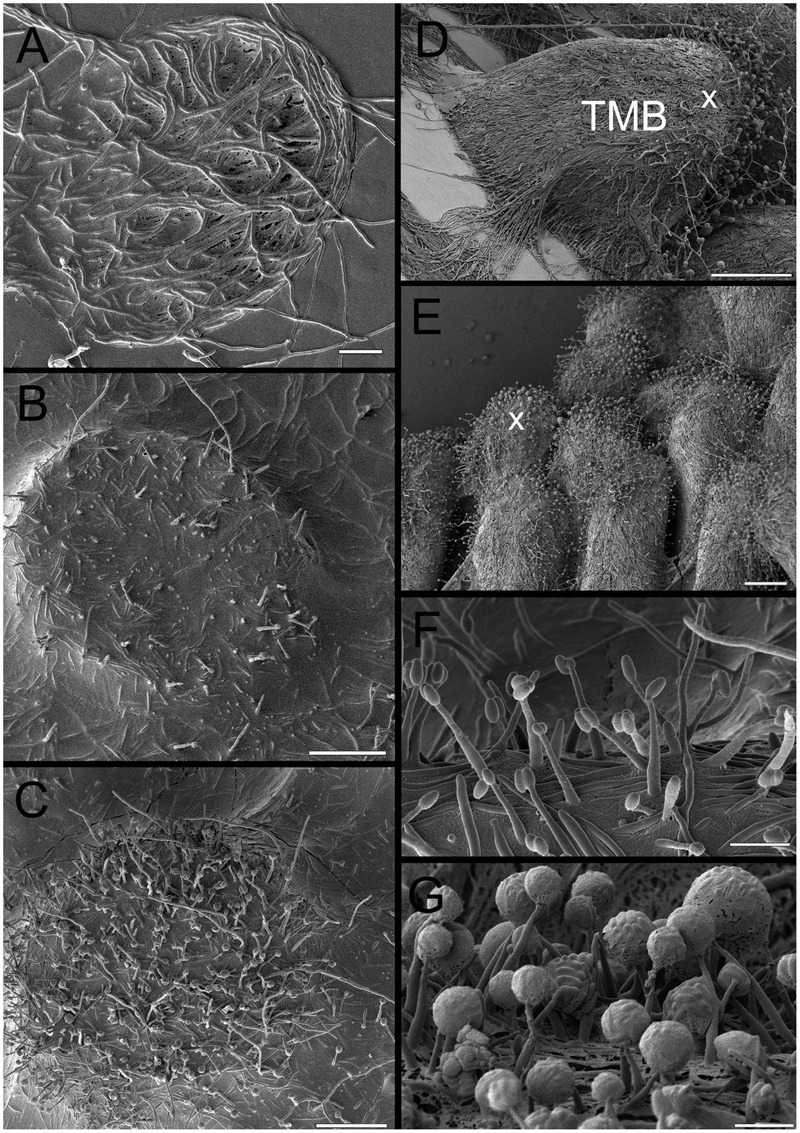
Overview of cryo-SEM image of developing TMB. **(A)** Trapping mycelia in a 180 μm diameter well after 2 days. **(B)** Growth out of the well after 3 days with limited dehydration prior to imaging, emphasizing the high degree of hydration. **(C)** As **(B)**, but at a slightly more advanced stage and with more removal of water revealing hyphae and some phialides. **(D)** Mature TMB after 8 days showing most phialides at the apex (x). **(E)** As **(D)** but showing multiple TMB. **(F)** Phialides after 3 days. **(G)** Close-up of phialides at apex of the TMB after 8 days. Note the large spherical clusters containing large numbers of conidia. Scale bar indicates 40 μm for **(A**–**C)**, 140 μm for **(D)**, 60 μm for **(E)**, and 5 μm for **(F,G)**.

TMB were compared to hyphal growth on agar by SEM (**Figure 5**). SEM confirmed the conclusions drawn from light microscopy: reduced branching was observed in the TMB compared to growth on agar or unstructured PAO (**Figures 5, 6A**). The development of TMB on MCC180.10 was monitored by microscopy with epi-illumination, SEM and cryo-SEM over a period of 10 days (**Figure 7**). The development path was the same whether the colony developed from conidia or hyphal entry, and increasing the inoculum of conidia from an average of 10 to 1000 CFU per well simply increased the speed of development. Hyphae readily crossed the walls dividing the wells, and then became trapped, generally following the curve of the wall within the well.

### Passive Uptake of Dyes

To investigate the uptake of dyes, the *Acremonium* isolate was grown on MCC, PAO or directly on ASWCLN agar with a variety of colored dyes in the agar. It was notable that dye uptake did not occur in early-stage growth (0–4 days) but initiated when the hyphae started to grow out of the MCC wells, coinciding with the time that the hyphae organized into vertical bundles. Despite a wide variety of chemistries and molecular weights, all dyes tested (nigrosine, dextran blue, a variety of food dyes) were incorporated into the *Acremonium* TMB cultured on MCC180.10. No dye uptake was observed for culture on agar or PAO. This was also the case for the fluorescent dyes fluorescein and rhodamine; the latter was used to quantify dye incorporation into TMB. The uptake of rhodamine was 50- to 1000-fold more efficient on MCC180.10 than on PAO or agar (**Figure 6B**). To investigate any role of active transport in dye uptake, a series of cultures were grown on MCC180.10 for 4 days and then transferred to a new medium containing various metabolic inhibitors at a concentration above the MIC, plus rhodamine and the cell-permeant, fluorogenic DNA stain Syto9 as a control. Rhodamine uptake was not inhibited by metabolic inhibitors (**Figure 6C**), suggesting that the dye was not being actively transported. Further, microscopy of these structures indicated that rhodamine dye uptake was largely extracellular. Dye uptake appeared to be by diffusion along the TMB. However, it was notable that if the TMB were submerged in liquid growth medium the dyes only leaked slowly. This suggests that within the TMB there is a local environment that retains solutes that do not equilibrate rapidly with the bulk phase.

### Physical Factors Triggering TMB Development

A series of microhabitats and physical environments were created to investigate what features were important in triggering the observed TMB.

(1) *Surface roughness.* As described above, culture on PAO alone was insufficient to trigger dye absorption. These experiments were repeated using PAO of 30 to 40% porosity, 200 nm average pore diameter, with different levels of roughness varying from 10 to 500 nm. In all cases, the result was the same: increasing the roughness of the surface did not trigger TMB or significant uptake of rhodamine.
(2) *Pore size.* Two different pore sizes of PAO, 20 and 200 nm in diameter, both with porosity 30–40%, were tested for dye uptake and TMB formation. In the absence of other structures (edges, wells) no TMB or rhodamine uptake was triggered.
(3) *Surface chemistry.* Surface treatments of PAO did not trigger MCC and modification of MCC with platinum or poly-L-lysine had no significant effect.
(4) *Compartment geometry on PAO and agar.* Microwells from 50 to 500 μm in diameter triggered TMB. Below that threshold individual hyphae penetrated microwells and the fungus grew on the surface but without the patterns of wells strongly influencing the hyphal structure, and with no formation of bundles nor uptake of dyes. Compartment spacing did not influence TMB formation: for MCC180.10VAR TMB development occurred regardless of whether the compartment was from 20 to 0.5 mm from the nearest TMB. However, TMB developing away from other TMB tended to grow horizontally rather than vertically. Deployment of the developed Ordyl photoresist (the wall material from the MCC180.10) directly on agar suggested that compartment geometry was not sufficient alone to trigger TMB.
(5) *Abrasion geometry on PAO.* Cuts from 5 to 20 μm deep and 50 to 300 μm wide within the PAO, creating channels with a porous base, were made with razor blades. Despite supporting growth and having walls (albeit porous rather than the impermeable walls on the culture chips) did not support the formation of TMB.
(6) *Wall height of wells.* It was possible to create culture areas 180 μm in diameter with a wall height of 0, 10, or 40 μm (0 is effectively a 180 μm circle of PAO with the surrounding area masked without protruding through to the upper side). Wells, i.e., growth areas with a physical wall, were created using different thicknesses of photosensitive films. Unfortunately, only a limited range of wall heights could be created due to the availability of materials. However, it was possible to deduce from these experiments that a degree of physical confinement was required, with MCC180.10 and MCC180.40 creating similar TMB.

### Development of Phialides

**FIGURE 8:**
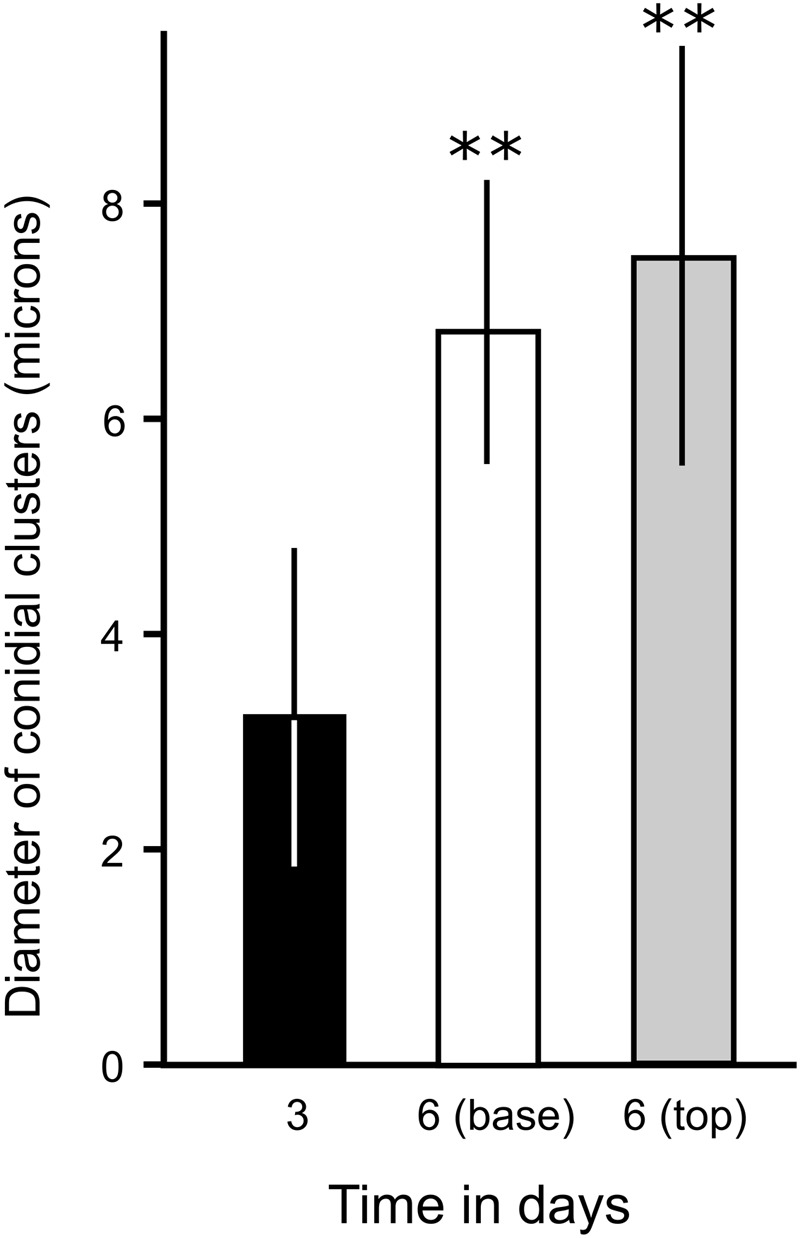
Quantification of the enlargement of conidial clusters of phialides during TMB development on MCCs. The diameter of phialides was assessed from cryo-SEM micrographs of developing TMB after 3 days (when the TMB are only just growing out of the microwells of MCC180.10) and after 6 days (measuring at the base and tip of the TMB). *n* = 20; mean ± SD. ^∗∗^Indicates a significant difference in the mean diameter of conidial clusters after 6 days (either at the base or tip of the TMB) compared to culture on MCC for 3 days (Student’s *t*-test, *P* < 0.05). No significant difference was found in the mean diameter of clusters, comparing the tip or base of the TMB after 6 days (*P* > 0.5).

The development of phialides was monitored during the growth of TMB by SEM (**Figures 5, 7**). Phialides appeared from 3 days after incubation both on MCC180.10, where TMB developed, and on PAO, for which hyphal development was in low, flat networks. It was notable that after 6–8 days, with full development, both the size and distribution of the phialides had changed. After 6 days, the phialides were predominantly at the apex of the TMB with others at the base. Both groups were of similar size (**Figure 8**) but significantly larger than after 3 days. Phialides were largely absent from most of the TMB between apex and base. Observation by low-powered microscopy suggested that the phialides were being generated after 3–4 days as the TMB became organized in the wells of the MCC180.10 and then were pushed upward. This implies that the growth of the TMB was subapical.

### Tracking the Development of TMB with Dye Particles

**FIGURE 9:**
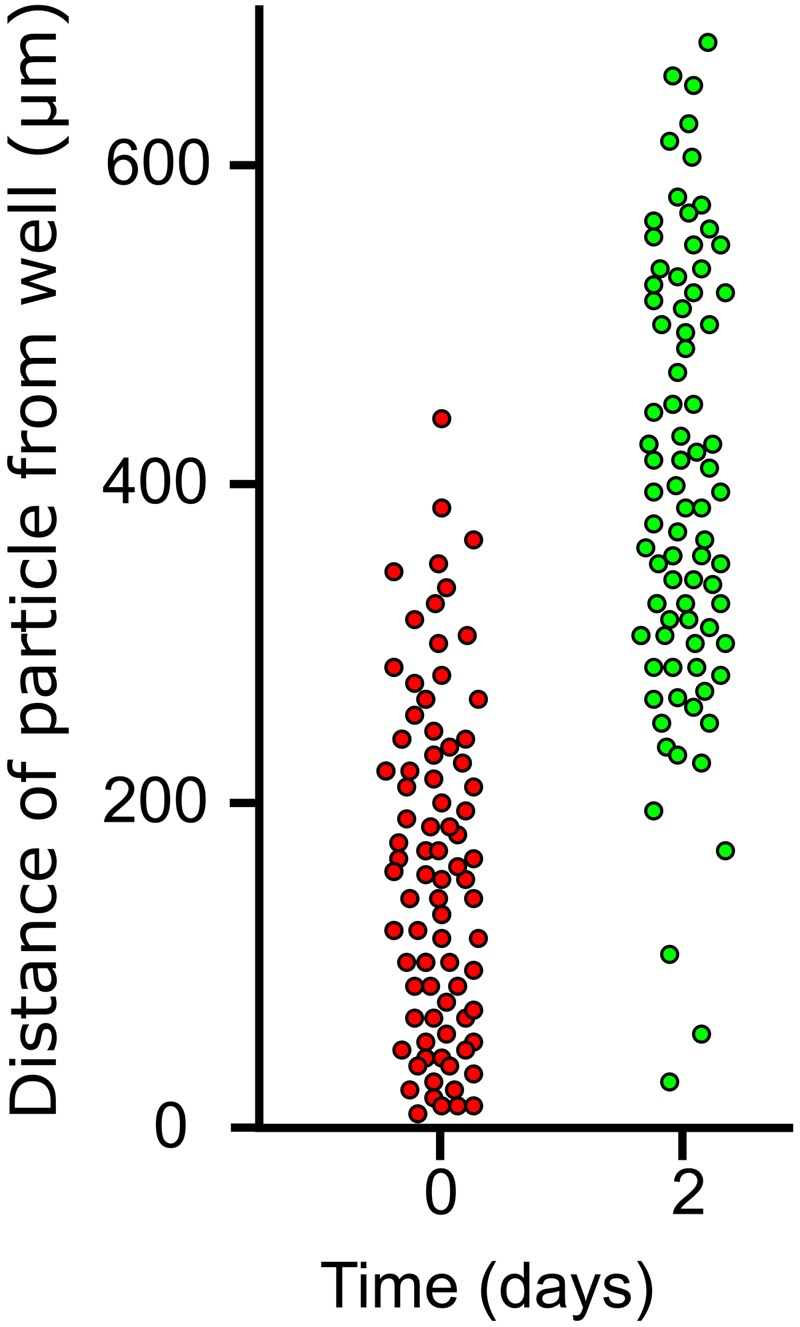
Distribution of dye particles on lateral TMB during growth. Laterally growing TMB emerging from wells of an MCC180.10 were dusted with poorly soluble dye particles after 5 days development and the positions (distance from well) of 100 particles on 20 TMB were measured. Two days later the same 20 TMB were assessed and the particle positions measured for a second time.

To test the idea that TMB growth was subapical, distribution of dye particles on horizontally growing TMB on MCC180.10VAR was measured immediately after addition and after 3 days growth (**Figure 9**). After 3 days, very few dye particles were found attached to the TMB within 200 μm of the well. Further, the standard deviation of the particle distribution only increased slightly, comparing the day of application with 3 days later. These observations suggest that the bulk of new growth occurs toward the base of the MCC.

### Hyphal and TMB Spread on MCC

**FIGURE 10:**
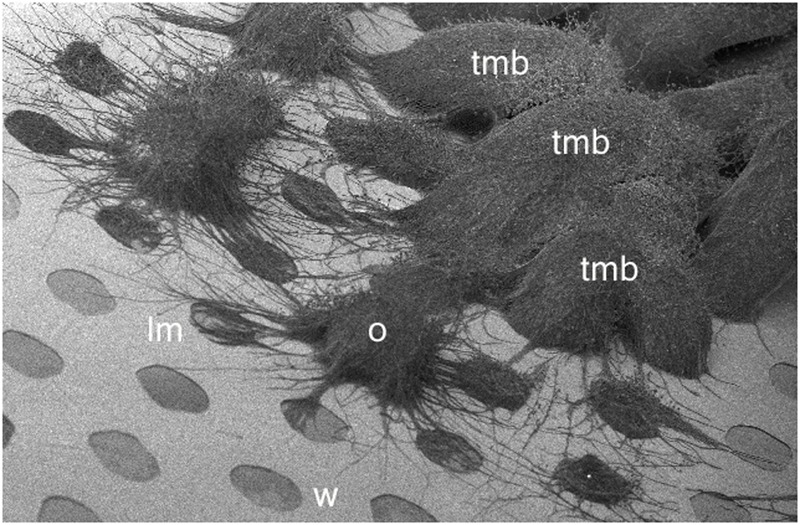
Cryo-SEM image of developing TMB on an MCC180.10 after 7 days. W, well (180 μm diameter); lm; lateral mycelia invading new wells; O, organizing tmb; mature TMB resulting from the initial inoculation and in most cases growing from one well and then into an adjacent well forming an arched hyphal bundle.

When hyphae were inoculated into the well of an MCC180.10 using a toothpick a TMB developed within 2 to 5 days. As the TMB emerged from the well, limited numbers of lateral hyphae (6–20 per TMB) were observed spreading over the MCC walls in all directions (**Figure 10**). After 3 days, most of the surrounding 180 μm diameter compartments, each 160 μm from the inoculation well, contained 1 to 5 hyphae. After 8 days the spread of TMB to the six adjacent wells was scored. In a minority of cases (6/100) the TMB failed to grow into any other well. In the majority of cases (92/100) only one adjacent well was invaded by the TMB. Only in rare instances (2/100) was more than one well spread to. These data suggest that the TMB forms a coherent and stable structure that commits to a specific direction rather than spreading in all directions or readily splitting to pursue multiple objectives.

These experiments were repeated using MCC180.10 in which the pores in the PAO at the base of some of the wells (surrounding the inoculation point) were blocked by applying graphite adhesive to the underside of the MCC. This treatment prevented the upper surface of the well from wetting when the MCC was placed on agar, but the adhesive did not fill the well. The graphite was effective in blocking the well, as it prevented a fluorogenic dye (Syto9, added to the agar underneath the MCC) from staining the hyphae in the MCC wells, this process occurring rapidly in unblocked wells. Targeted inoculations were made so the developing lateral hyphae and subsequent TMB had the “choice” of spreading to adjacent wells that were either blocked or unblocked. In observations of 50 such experiments, blockage of wells did not inhibit the entry of lateral hyphae after 5 days. The probability of a mycelium entering an adjacent well was 0.87 for blocked wells and 0.95 for unblocked wells, indicating an unbiased spread in all directions. Indeed, lateral hyphae could spread up to 2 mm across the walls of blocked MCC from a TMB and generate phialides at that distance. However, scored after 8 days, TMB did not develop in blocked wells from adjacent wells, despite the presence of limited numbers of lateral hyphae in those wells. The probability of a mycelium developing within a blocked well into a mature TMB was 0.04 (2 instances in 50 trials) and 0.36 (18 instances) for an unblocked well. In the two cases where a TMB spread to a blocked well, the TMB did not develop further. In the 18 cases where TMB spread to unblocked wells, 12 spread further, to a third unblocked well. In summary, lateral hyphae appeared to explore the immediate environment in all directions but were topologically sensitive in that they entered and/or became trapped in the microwells of the MCC180.10. However, the TMB could discriminate a blocked from an unblocked well. We conclude that the entry into wells of lateral hyphae is independent of the nutrient supply or water availability in the destination, but that the extension of TMB from one well to an adjacent well is strongly influenced by the condition of the target.

### Culture on Sponge Surfaces

The surface of a dead sponge, not supplemented by nutrients, was sufficient to support the growth of the *Acremonium* isolate at a similar rate to growth on MCC. Hyphal bundles formed when the fungus was growing on the porous textured surface, resembling the TMB on MCC. Growth was exclusively outward, i.e., from the sponge surface into the air: no hyphal penetration into the pores of the sponge was detected.

## Discussion

Marine sponges represent unique, structured ecosystems. Little is known about the interaction of fungi with sponges (Yarden, 2014). More information exists for bacterial–sponge interactions, which can be complex and may result in the production of novel, bioactive secondary metabolites that cannot be synthesized by either party in isolation. Among other fungi, *Acremonium* spp. have been isolated from sponges in locations as diverse as the Mediterranean, North Atlantic and Pacific Oceans (Abraham et al., 1955; Paz et al., 2010; Perdomo et al., 2011). However, the level of adaptation to any such hosts has not been investigated. Indeed, the degree of adaptation to the marine environment is often unclear as not all such isolates are halotolerant (Yarden, 2014).

Cultivation in new ways may lead to the discovery of new microorganisms, or prompt already known microbes to change their growth behavior. Typically, cultivation strategies focus on optimizing nutritional components and other chemicals such as signaling compounds. However, it is well known that other factors can be important for growth and development, including light, temperature, and the physical environment. In this work, a microengineered cultivation chip, consisting of an array of microwells with a porous ceramic base, was used to isolate an *Acremonium* sp. This fungus was found to be a novel species, with very different growth morphology compared to growth of the same fungus on agar.

Hyphal bundles (TMB) formed on the chips, with their width specified by the well diameter. A wide range of microenvironments were fabricated to try and determine the conditions by which TMB were generated. Microwells 50 to 500 μm in diameter and 10 μm deep triggered the formation of TMB. These observations suggest that physical confinement may play a part in generating TMB. Despite emerging from wells of limited height (10 μm) TMB could extend at least 0.5 mm whilst maintaining a diameter very close to that of the microwell. The material forming the base of the well was also important: agar at the base of microwells did not trigger TMB. PAO was required at the microwell base, but PAO alone was not sufficient to trigger TMB development. Taking these observations together with SEM observations leads us to suggest the following stages in the development of the TMB:

(1) Germination of spores within wells, or entry and trapping of hyphae within wells.
(2) In either case, inoculation of a microwell leads to hyphal growth filling the wells. The density of inoculation simply affects the rate at which TMB formed; a single spore can initiate a TMB. During this period phialides are generated.
(3) As the hyphae grow out of the wells there is *ad hoc* organization of most hyphae into vertical bundles. A smaller number of hyphae are not incorporated into the TMB but grow laterally in all directions, apparently without bias.
(4) Once the TMB grows out of the microwells, this assembly of aligned hyphae is stable and conforms to the diameter of the microwell. Long-term changes in gene expression appear to be triggered by early confinement of hyphae in microwells with PAO bases. This includes suppression of side branching, which when growing on agar or unstructured PAO is a common event. Phialides generated early are pushed away from the wells and remain localized around the tip of the TMB. The phialides continue to develop, so the ones at the tip are larger. The TMB are highly hydrated structures, allowing small molecules to diffuse rapidly from the base to the tip.
(5) TMB can grow vertically, or laterally. In the latter case entry into adjacent wells occurs without splitting and requires the target well to be unblocked (hydrated with nutrients). In this way TMB can propagate laterally, utilizing multiple nutrient sources. TBM can continue to grow to nearly 1 cm in length.

We found that organization of TMB is highly coherent, using MCC180.10VAR chips to provide good spacing between TMB in adjacent wells. At the edge of an expanding TMB a small number of hyphae grew in all directions, over the surface of the wall, without discriminating between “good” and “bad” wells. However, the mature TMB only moved in one direction, suggesting that the TMB are highly stable structures and not easily divisible. Further, the TMB had a strong tendency to end up in favorable environments, i.e., unblocked, hydrated wells with nutrients at the base. This suggests a superior sensory capacity, possibly using the lateral hyphae to explore the local environment before risking a larger number by committing a TMB (and therefore a large number of hyphae) to any given direction. There are a number of possible functions for TMB. They may create a locally favorable environment to exclude competitors or resist predators. The ability of TMB to accumulate dyes suggests a role in the accumulation, and even transport, of low molecular weight compounds, including nutrients. The dense array of parallel hyphae may act as capillaries in this respect. It is also conceivable that the TMB position spores in an optimal position for dispersal. There was a tendency of mycelial bundles to grow out of marine sponges rather than penetrate inward; this would push the maturing phialides out into the surrounding marine environment. The link between TMB and other parallel mycelial structures, such as rhizomorphs (which are also known as mycelial cords) is unclear (Moss, 1987). As far as we are aware, the physical conditions for the creation of rhizomorphs are unclear. However, the ability to create precisely calibrated confinement of mycelia should allow such links to be explored in the future.

Microfabricated environments offer considerable potential in tailoring the growth of individual microorganisms or of microbial communities. Within this study we varied many parameters including geometry of the growth well, the density of microbial microcolonies, surface chemistry, nutrient access, porosity and texture of the growth matrix, and wetness. Many of these properties can be varied precisely and within a single experiment, often within a single culture chip, to enable a systematic exploration of the interaction between environment and microbe. For example, it is possible to assay many aspects of microwell geometry on the micron scale and that of the PAO on the 10–500 nm scale. Here, we have exploited a porous ceramic-based culture chip to both isolate a novel fungus and reveal physical triggers for unexpected morphological complexity. Further advantages include economical use of expensive reagents and the facility to add and remove compounds by moving the chip. Other cultivation approaches include gel encapsulation, providing near-natural culture conditions. We expect more complex microbial communities to be approached in this way in future. It also appears likely that commercial applications will emerge from the exploitation of TMB or other forms of “mycelial engineering.” Engineering mycelial structures may allow the development of new materials as mycelial structures are directly recycled into building materials or used as the basis to design structures (i.e., by the process of biomimetics) made out of other materials^1^. Further, engineered mycelia may produce different secondary metabolites compared to other growth methods and thus facilitate drug discovery from fungi. Lastly, if TMB are relevant to the invasion of environments or host tissues by pathogenic fungi then they may be good targets to screen for inhibition by antifungal agents.

## Author Contributions

LC performed fungal culture and DNA sequencing, AY analyzed phylogenetic data, MG performed electron microscopy, JD designed experiments, performed electron microscopy, and wrote the manuscript, CI designed and performed culture chip experiments and optical microscopy and wrote the manuscript.

## Conflict of Interest Statement

CI is involved in the research and design of commercial microfabricated cultivation chips. The other authors declare that the research was conducted in the absence of any commercial or financial relationships that could be construed as a potential conflict of interest.

## Footnotes

1 http://www.corpuscoli.com/projects/mycelium-design/
